# Protocol for fluorescent false neurotransmitter live imaging of dopamine release dynamics from individual synapses in acute brain slices

**DOI:** 10.1016/j.xpro.2026.104773

**Published:** 2026-08-06

**Authors:** Patrick Cottilli, Eugene V. Mosharov, Michael J. Devine, David L. Sulzer

**Affiliations:** 1 Mitochondrial Neurobiology Laboratory, The Francis Crick Institute, 1 Midland Road, London NW1 1AT, UK; 2 Department of Clinical and Movement Neurosciences, UCL Queen Square Institute of Neurology, University College London, London WC1N 3BG, UK; 3 Columbia University, New York, New York, NY 10027, USA; 4 New York State Psychiatric Institute, New York, New York, NY 10032, USA

**Keywords:** Microscopy, Neuroscience, Molecular/Chemical Probes

## Abstract

Striatal dopaminergic (DA) axons have many *en passant* boutons with synaptic vesicles, but only a fraction display exocytosis. Here, we present a protocol for live imaging of murine DA axons using fluorescent false neurotransmitter 200 (FFN200), a vesicular monoamine transporter 2 (VMAT2) substrate accumulated by and released from DA synaptic vesicles, providing spatial and temporal kinetics of exocytosis. We describe steps for preparing mouse acute brain slices, loading slices with the FFN, acquiring images with 2-photon microscopy, and analyzing the data.

For complete details on the use and execution of this protocol, please refer to Hwu et al.^1^

## Highlights

• Instructions for the preparation of acute brain slices
• Steps for live imaging of FFN200 at dopamine axons with 2-photon microscopy
• Guidance on a pipeline for data analysis from raw images to data visualization

## Abstract (editor highlights)

Publisher’s note: Undertaking any experimental protocol requires adherence to local institutional guidelines for laboratory safety and ethics.

## Graphical abstract

**Figure:**
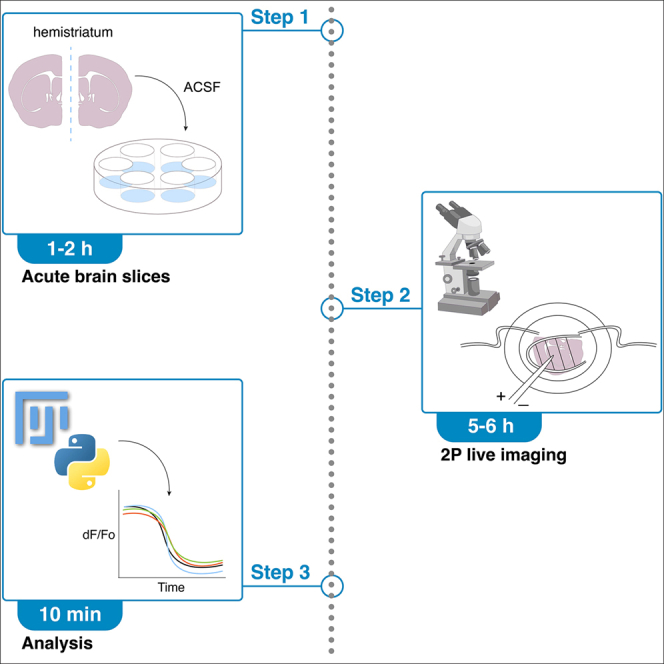

## Before you begin

Dopamine (DA) signaling in the brain is a critical regulator of learning, behavior, emotion and motor control. Disruption of DA signaling plays a key role in a wide range of neurological and psychiatric conditions including Parkinson's disease, schizophrenia and addiction. However, the precise spatiotemporal dynamics of DA release are poorly understood. Previous work has demonstrated that DA boutons are highly heterogeneous: only a small fraction (∼20%) of striatal DA boutons are capable of DA release.^2^ Furthermore, only a minority (∼30%) of DA boutons contain presynaptic proteins required for DA release.^3^ Therefore, techniques are needed that can resolve dopamine release dynamics at the level of individual synapses.

Fluorescent false neurotransmitter 200 (FFN200) is a substrate for vesicular monoamine transporter 2 (VMAT2), which allows its selective uptake by monoaminergic synaptic vesicles.^2^^,^^4^ Although the compound can potentially label recycling endosomes, such labelling is negligible in striatal coronal brain slices since vesicle recycling is minimal due to the absence of intrinsic activity in this brain preparation. Consistent with this, 86% of FFN200 puncta colocalizes with the synaptic marker synaptophysin.^2^ Moreover, the number of FFN200 puncta decreases significantly in striatal slices from VMAT2 hypomorph mice, as well as in wild-type mice slices incubated with a VMAT2 inhibitor.^2^ Due to its high selectivity, FFN200 is used to visualize monoamine exocytosis in brain tissue. It was used for the first time to demonstrate DA release dynamics at individual synapses, and the presence of silent DA synapses.^2^ FFN200 is commercially available and does not require the use of transgenic animals or protein expression vectors. Following a short incubation with the tissue, the compound can be readily imaged at 350/450 nm excitation/emission maxima. Due to FFN200 emission in the blue spectral region, it can be combined with other commonly employed fluorescent labels (eGFP, TxRed, etc.).

Here we describe a full protocol for the implementation of FFN200 imaging, including preparation and loading of tissue with FFN200, as well as image acquisition and analysis. We include full data analysis code in open-source programming languages to enable widespread adoption in any laboratory interested in DA neurotransmission kinetics.

### Innovation

Live imaging of DA release is essential to determine the spatiotemporal regulation of this neurotransmitter. Multiple approaches have been developed to detect DA release (extensively reviewed in^5^), however these typically lack sufficient spatial resolution to resolve individual DA boutons. For example, genetically encoded postsynaptic sensors (e.g., dLight1^6^^,^^7^ and GRAB_DA_^8^^,^^9^) are commonly used to measure released neurotransmitter, but they do not label the sites where DA accumulates and is released from, and so do not detect fundamental features of exocytosis, such as silent DA boutons or release kinetics of individual DA boutons. VMAT2-pHluorin^10^ provides a means to measure presynaptic changes, but so far has been unable to detect silent DA boutons, and to our knowledge has not been successfully used in the intact mammalian brain or in slice preparations. Moreover, these sensors require genetic approaches, potentially altering physiological synaptic activity. Non-genetically encoded strategies to detect DA release include a 2D nanofilm,^11^ and a nanosensor paint^12^; both have demonstrated silent DA synapses, but in dissociated cultured neurons rather than tissue, and they are reliant upon sensor technologies that require multiple complex steps. In contrast, FFNs allow live imaging of individual DA boutons in acute brain slices without additional protein expression, enabling a readout of DA release kinetics. Here we describe an analysis protocol with open-source code, which is available on GitHub. While this manuscript describes the use of FFN200, additional FFNs can be used with similar approaches.

### Institutional permissions

Animals were maintained according to the National Institutes of Health guidelines in Association for Assessment and Accreditation of Laboratory Animal Care (AAALAC) accredited facilities on a 12:12 h light/dark cycle with food and water *ad libitum*. All experimental procedures were approved by the Columbia University Institutional Animal Care and Use Committee and followed guidelines established in the NIH Guide for the Care and Use of Laboratory Animals.

### Warming up 2P Pockels cells

**Timing: > 3 h**

If your 2-photon (2P) microscope has Pockels cells, they need to be warmed up to ensure stable laser power. Start up the 2P microscope, keep the shutter for sample exposure closed, but open the shutter to irradiate the Pockels cells with a low laser power for at least 3 h. Set the 2P laser wavelength to 740 nm, which will be used for the actual experiment.

**CRITICAL:** If Pockels cells are not pre-warmed, laser power may drift throughout the experiment, affecting imaging reproducibility.

***Note:*** The irradiation timing might change depending on your Pockels cells. It is advisable to measure the laser power intensity throughout this time and obtain the time at which the laser power stabilizes.

### Setup for brain extraction and slicing

**Figure 1:**
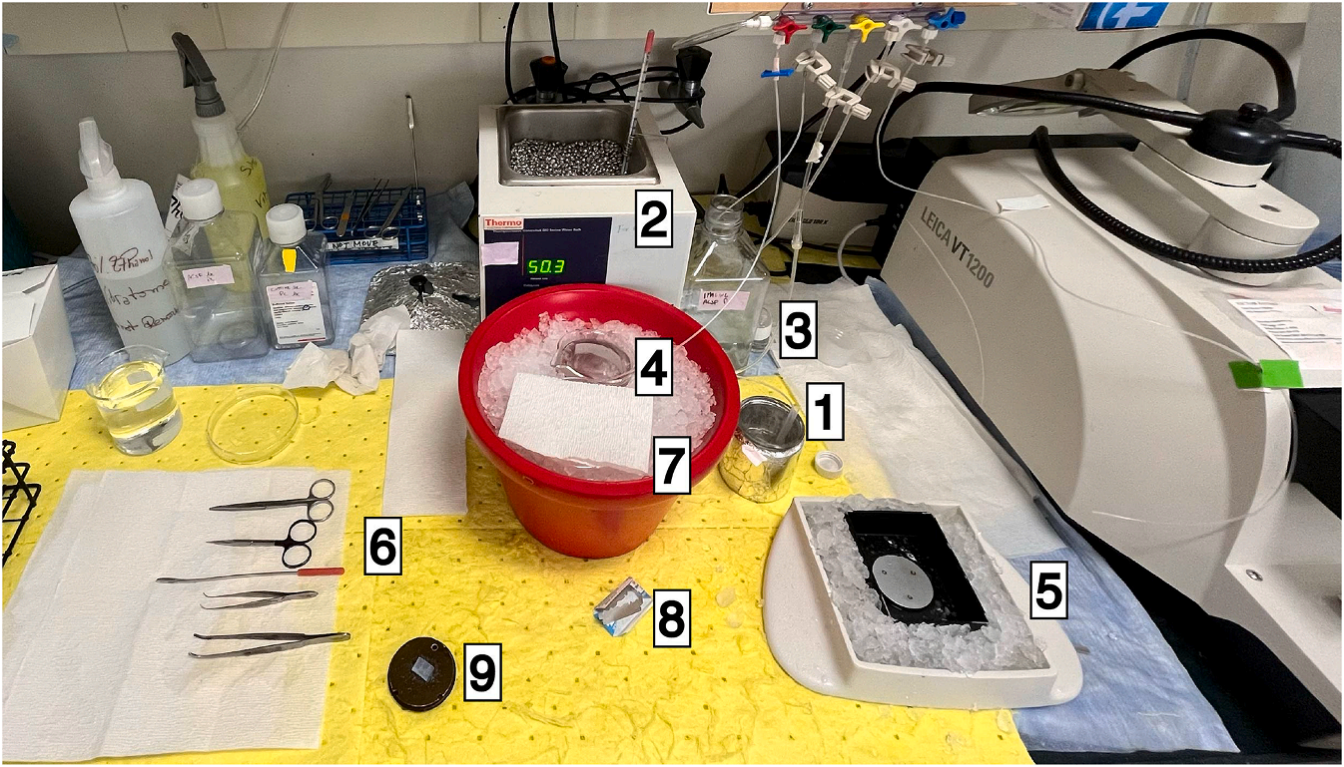
Setup for acute brain slice preparation (1) Slice holder. (2) Heating beads. (3) Bijou vial. (4) Beaker with cutting solution. (5) Vibratome slicing tray. (6) Dissection tools. (7) Petri dish with tissue on top. (8) Razor blade. (9) Cutting chamber.

**Timing: 30 min** (see Figure 1 for reference)

Ahead of time: 5 min.

1. Dissolve FFN200 in DMSO at 20 mM final concentration.
  a. Aliquot the stock in 5 μL and store at −20°C for up to one month.
2. Prepare artificial cerebrospinal fluid (ACSF); 500 mL is sufficient for one brain.
  a. Add enough ACSF to cover the slice holder and place it in a water bath at 37°C.
    ***Note:*** Heating beads can be used at 50°C. The higher temperature is needed because beads transmit heat at a slower rate.
  b. In a Bijou vial, add 4-5 mL of ACSF, and place it heating together with the slice holder.
    ***Note:*** This vial is used later to incubate slices with FFN200.
3. Prepare cutting solution; 250 mL is sufficient for one brain. Place the beaker with the cutting solution in an ice bucket.
4. Bubble a gas mixture of 95% O_2_ and 5% CO_2_ (carbogen) through the remaining ACSF, the slice holder, the Bijou vial for FFN200 labeling and the cutting solution to saturate solutions with oxygen and to buffer the pH with dissolved CO_2_.

**CRITICAL:** Bubble the media with carbogen for at least 15 min before starting the brain extraction.

5. Mount the vibratome slicing tray, cover it with the plastic cap the company provides, and fill the surrounding space with ice.
6. Place all necessary instruments for the extraction and cutting nearby.
  a. Two scissors (one large and one fine with pointed ends).
  b. Two bent forceps (one large and one fine).
    ***Optional:*** A spatula with a small end to scoop up the brain.
  c. Instant drying superglue.
    ***Optional:*** Better results are obtained with superglue equipped with a brush.
  d. Open a 20 cm petri dish and put upside down one of the halves.
7. If using disposable double-edged razor blades, break one in half and mount one half in the vibratome. Place the other half together with the other instruments for preparing the brain prior to slicing. Otherwise, use two different razor blades.

## Key resources table

**Table:** 

REAGENT or RESOURCE	SOURCE	IDENTIFIER
**Biological samples**		

Mouse derived acute brain slices	New York State Psychiatric Institute	N/A

**Chemicals, peptides, and recombinant proteins**		

NaCl	Thermo Scientific	7647-14-5
KCl	Sigma-Aldrich	P3911
NaHCO_3_	Thermo Scientific	144-55-8
CaCl_2_	Sigma-Aldrich	E9884
MgCl_2_ x 6H_2_O	Thermo Scientific	7731-18-6
NaH_2_PO_4_ x H_2_O	Sigma-Aldrich	10049-21-5
Glucose	Sigma-Aldrich	67528
Sucrose	Sigma-Aldrich	57-50-1
FFN200	Tocris	Cat#: 5911

**Deposited data**		

Python Code	This paper	Github: https://github.com/MDevineLab/FFN200-live-imaging Figshare: https://doi.org/10.25418/crick.32731767
FIJI Code	This paper	Github: https://github.com/MDevineLab/FFN200-live-imaging Figshare: https://doi.org/10.25418/crick.32731767
Sample data	This paper	Github: https://github.com/MDevineLab/FFN200-live-imaging Figshare: https://doi.org/10.25418/crick.32731767

**Experimental models: Organisms/strains**		

C57BL/6, P60-90, mixed sex	The Jackson Laboratory	RRID:MGI:2159769

**Software and algorithms**		

Master 8 – Version 1.9.2.0	A.M.P.I.	N/A
Prairie View – v.5.8	Bruker	N/A
FIJI – v.2.14.0/1.54f	Schindelin et al.^11^	https://imagej.net/software/fiji/downloads
Python 3.11	Python	N/A
Prism 11	GraphPad	https://www.graphpad.com/

**Other**		

Prairie Ultima	Bruker	https://www.brukersupport.com/globalsearch/search?t=2
Coherent Chameleon Ultra II	Coherent	https://www.photonics.com/Products/Chameleon-Ultra-II-Laser/pr29201
40x Olympus LUMPlan FL N	Olympus	N/A
Pockels cells 350-80	Conoptics	N/A
Photomultiplier	Hamamatsu	https://www.hamamatsu.com/us/en/product/optical-sensors/pmt/pmt_tube-alone/side-on-type/R3896.html
Carbogen	N/A	N/A
Master 8	A.M.P.I.	https://www.ampi.co.il/master-8
ISO-Flex	A.M.P.I.	https://www.ampi.co.il/iso-flex
Tungsten concentric bipolar electrode	WPI	TM33CCINS
Bijou vial	starlab	E1412-0710
Vibratome	Leica	VT1200
Peristaltic pump	N/A	N/A
Slice Anchor (Harp)	N/A	N/A
Micromanipulator	N/A	N/A
Imaging perfusing chamber	N/A	N/A
Volumetric flask (1 L)	N/A	N/A
Volumetric flask (500 mL)	N/A	N/A
Two forceps	N/A	N/A
Two scissors	N/A	N/A
Superglue	N/A	N/A

## Materials and equipment

**Table:** Cutting solution

Reagent	Final concentration (mM)	Amount (g)
NaCl	10	0.584
KCl	2.5	0.186
NaHCO_3_	25	2.1
CaCl_2_	0.5	0.055
MgCl_2_ x 6H_2_O	7	1.423
NaH_2_PO_4_ x 2H_2_O	1.25	0.195
Glucose	10	0.9
Sucrose	180	30.8
ddH_2_O	N/A	Up to 500 mL
**Total**	**N/A**	**500 mL**

Store at RT for up to 2 weeks.

**CRITICAL:** Osmolarity must be 300±10 mOsm.

**Table:** ACSF

Reagent	Final concentration (mM)	Amount (g)
NaCl	125	7.305
KCl	2.5	0.186
NaHCO_3_	25	2.1
CaCl_2_	2	0.222
MgCl_2_ x 6H_2_O	1	0.203
NaH_2_PO_4_ x 2H_2_O	1.25	0.195
Glucose	10	1.8
ddH_2_O	N/A	Up to 1 L
**Total**	**N/A**	**1 L**

Store at RT for up to 2 weeks.

**CRITICAL:** Osmolarity must be 300±10 mOsm.

## Step-by-step method details

### Acute brain slices preparation and first FFN200 incubation

**Timing: 1 h**

1. Brain extraction.
  ***Note:*** This step should be done as swiftly as possible to maximize tissue viability.
  a. Euthanize the animal by cervical dislocation followed by rapid decapitation with the pair of sharp scissors.
  b. Remove the skin to expose the skull.
    i. Cut the skin from the midplane of the head to the middle of the skull.
    ii. Cut the tissue connecting each ear to the head.
    iii. Pull the skin towards the animal nose, exposing the skull.
  c. Insert one blade of the fine pointed scissors at the cut end of the head and make three cuts through the bone of the skull - one along the midline, and two on each side of the skull, as shown in Figure 2A.

**Figure 2:**
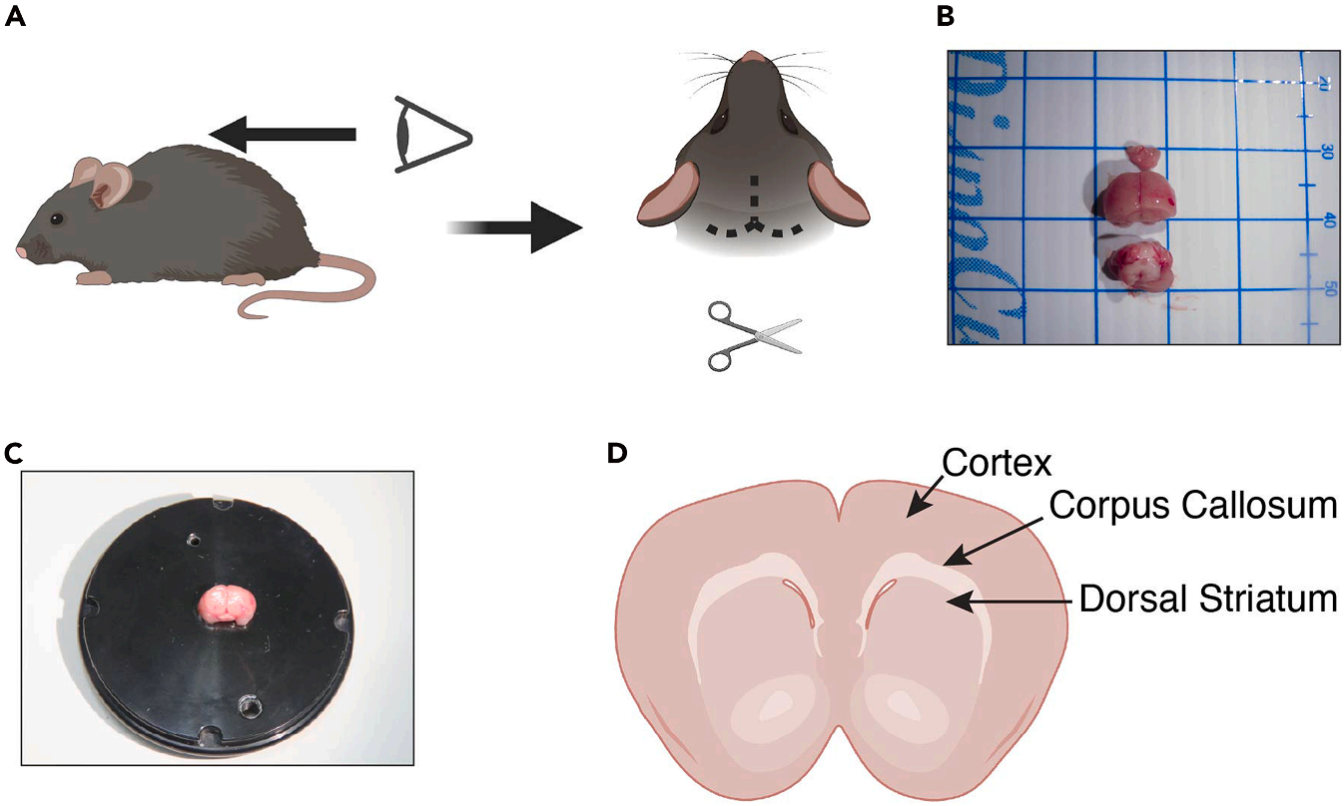
Diagram for mouse brain extraction and dissection (A) Schematics depicting skull opening and removal. (B) Dissected brain with removed olfactory bulbs (top) and cerebellum (bottom). (C) Mounted brain in the vibratome cutting chamber. (D) Schematics of the coronal section for imaging.
  d. Insert the closed fine scissors between the olfactory bulbs and the olfactory septum. Then firmly open the scissors. This will open the skull in half exposing the brain.
    ***Optional:*** If the user is still not quick at extracting the brain, the head can be placed in a petri dish with cold cutting solution. This will help keep the brain tissue healthier.
  e. With the fine forceps, remove any additional skull fragments covering the brain.
  f. Carefully scoop out the brain using the spatula.
  g. Place the brain on top of the upside-down petri dish half and cut off the olfactory bulb and the cerebellum (Figure 2B).
    ***Note:*** Make the caudal cut as straight as possible, so that the brain sits vertically in the vibratome.
  h. Apply superglue to the base of the cutting chamber and place the brain with the rostral side up (Figure 2C).
  i. Immediately fill the chamber with ice-cold cutting solution.
2. Acute brain slicing.
  a. Using high-speed settings on a vibratome (1.5 mm amplitude, 1 mm/s speed or higher, 250 μm thickness) slice and discard the brain till striatum is exposed (Figure 2D).
  b. Change the cutting settings to: 1.5 mm amplitude, 0.1 mm/s speed, 250 μm thickness. These parameters ensure less tissue damage and better slice preservation.
  c. When the first slice is ready to be collected, remove the slice holder and Bijou vial with ASCF from the heating source and keep at room temperature (20-25°C).
  d. About 6 slices per brain can be obtained. Slices can be maintained in a slice holder with carbogen bubbling for up to 5-6 h.

***Optional:*** Slices can be cut in half so that each hemi-striatum can be treated individually.

3. Incubate with FFN200.
  a. Add FFN200 to the Bijou vial at a final concentration of 10 μM. Cover the vial with aluminum foil to protect it from light.
  b. Incubate slices simultaneously or sequentially in the Bijou vial(s) with FFN200 for 30 min with constant bubbling with carbogen.

### 2P live imaging

**Timing: > 5 h**

Performing live imaging of acute brain slices incubated with FFN200.

**CRITICAL:** Bubble the media with carbogen at all times.

4. After 30 min incubation with FFN200, wash out the dye for 15 min by putting back the slice in the slice holder.
5. Transfer the slice to the imaging chamber and secure it with a ‘harp’ (Figures 3A and 3B). Let the imaging setup accommodate for 20-30 min. This ensures minimal mechanical drift during image acquisition.

**Figure 3:**
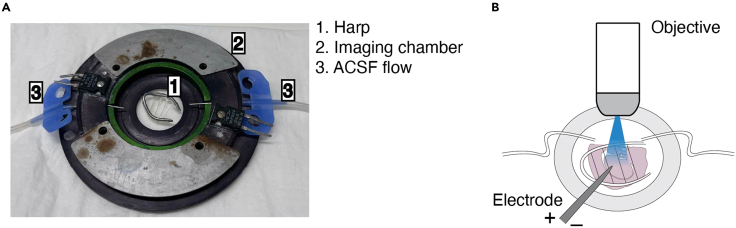
Imaging setup (A) Imaging chamber. (B) Schematics for imaging.
6. In the meantime, locate the area of interest (see schematic on Figure 2D) using eyepieces and brightfield illumination on the microscope.
7. Switch to 2-photon imaging with excitation laser at 740 nm, and emission filters of 470/60 nm.
8. Confirm that there is FFN200 signal in the blue spectra.
9. Place the stimulation electrode on top of the slice.

**CRITICAL:** Do not insert the electrode too deeply into the slice, because the electrical stimulation creates movement artefacts.

***Note:*** When the tip of the electrode is *touching* the slice, very gently jiggle the electrode; if the surrounding tissue is slightly moving, you have correctly placed the electrode.

10. Locate a region near the stimulation electrode to acquire the unstimulated control recording.

***Note:*** Image at a slice depth >= 20 μm to ensure recordings are performed in a layer of tissue undamaged by slicing. Typically, FFN200 staining can be observed at >50 μm depth, albeit with progressively decreasing brightness. We usually image at 20-50 μm from the slice surface.

11. Start the imaging timeseries at the following settings:
  a. 45 time points at 11 s interval. Total time > 8 min.
  b. Each time point comprises a 5-image z-stack with a step size of 0.5 μm.
  c. Pixel dwell time of 4.4 μs.
  d. 256x256 pixels images.
  e. Laser power: 2.1–2.2 mW.
  f. 800 HV photomultiplier.
  g. Approximately 22x22 μm^2^ region of interest.
  h. 8x digital zoom.
  i. 4 frames averaging.
12. Next, locate a new region at 50-100 μm from the tip of the stimulation electrode and repeat the recording, starting the electrical stimulation at the 20^th^ frame (biphasic square pulses: 15 Hz, 100 μA, 160 s, 2400 total pulses).

***Note:*** Imaging further than 100 μm from the tip of the electrode greatly reduces stimulation current density and the chances of observing FFN destaining.

**CRITICAL:** From our experience, electrical stimulation with biphasic pulses produce more consistent results than with monophasic (positive or negative) current pulses.

### Data analysis

**Timing: < 10 min**

**Figure 4:**
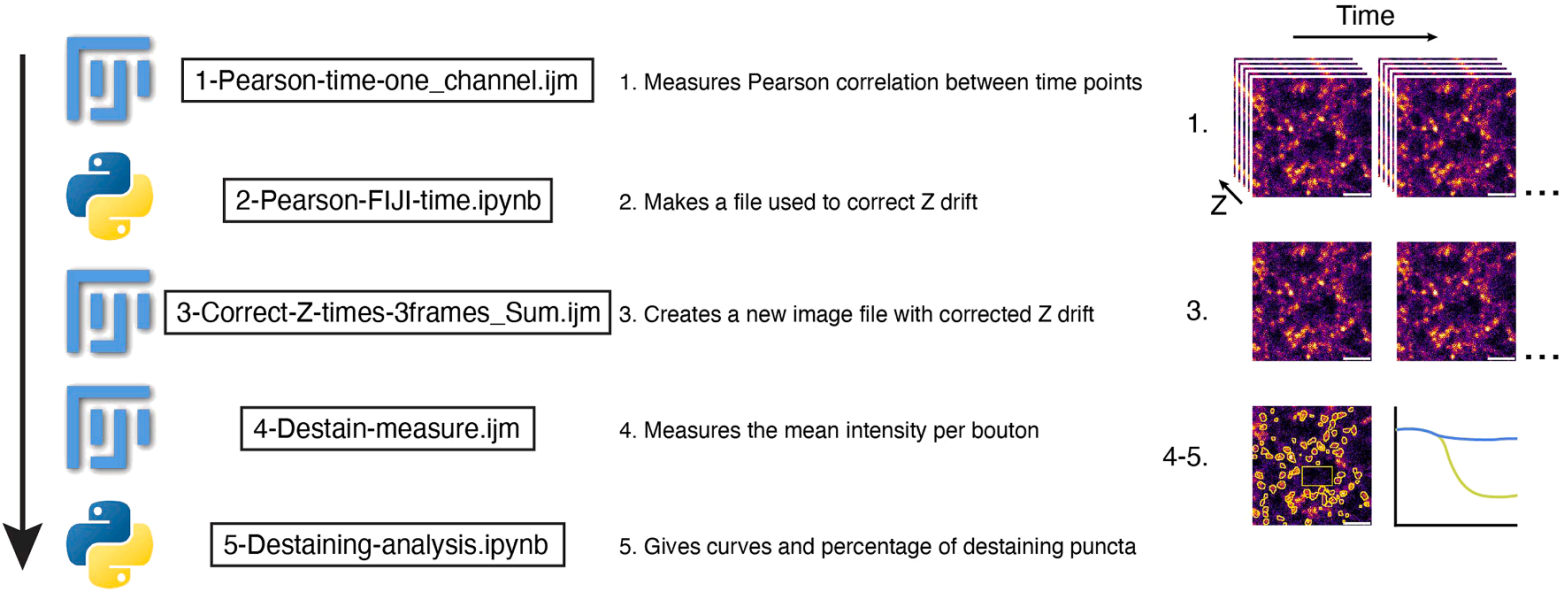
Code sequence and functions description

To analyze the images, we use FIJI^13^ followed by Python3 (either via terminal or Jupyter). Successful execution of each code chunk is concluded with “Command finished!” message. See Figure 4 for a schematic flow of the code.

***Note:*** All “.ipynb” files are only compatible with Jupyter. However, the code can be copied into a new file as “.py” and run through the terminal.

13. Open the “1-Pearson-time-one_channel.ijm” code in FIJI and run it.
  a. The code prompts you to open the image file.
  b. Confirm that the first half of the time series will be analyzed (Figure 5A).
    ***Note:*** Because the images are large, sometimes the computer can only analyze half of the images at once, and so the current code is adjusted for this situation.

**Figure 5:**
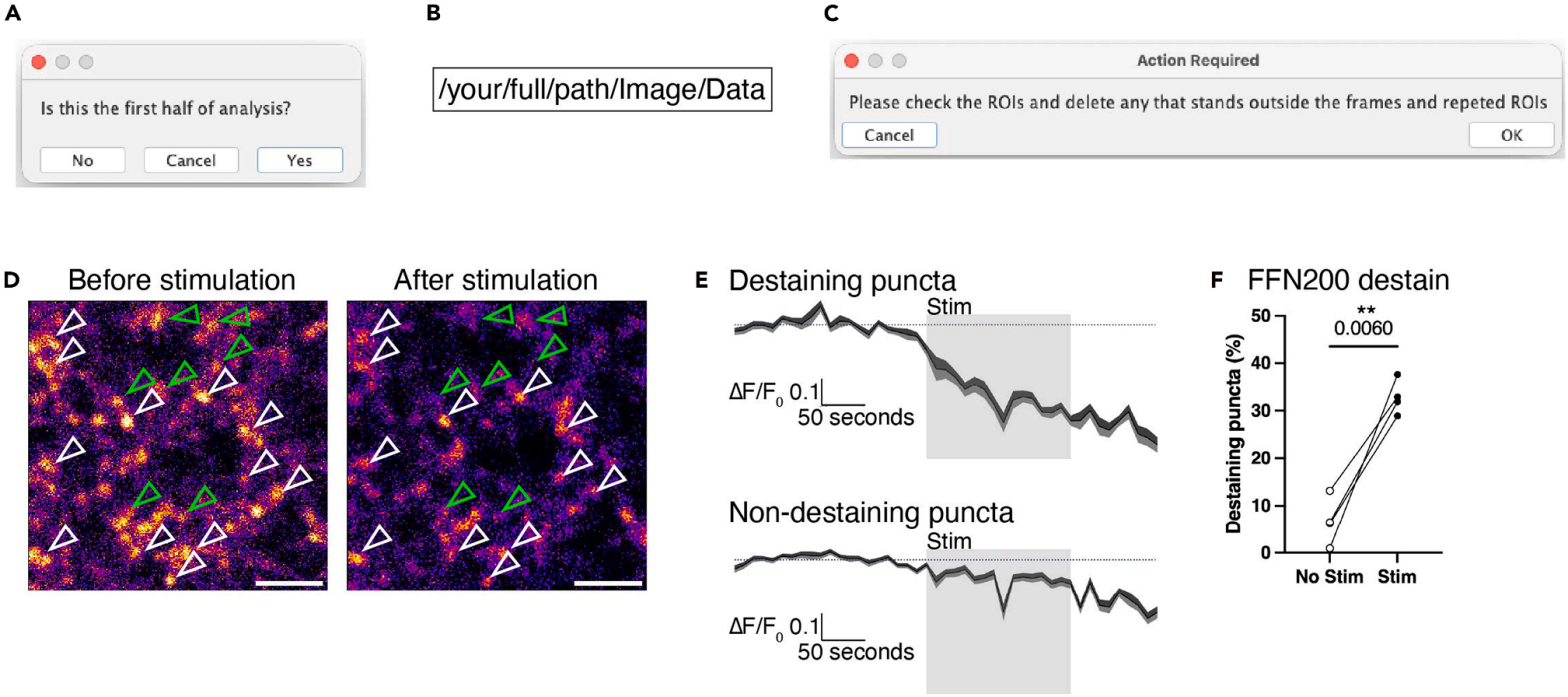
FFN200 live imaging in acute striatal slices (A) Snapshot showing the prompt from step 13. (B) Example path for the codes written in Python. (C) Snapshot showing the prompt from step 16. (D) Snapshots before and after electrical stimulation of a representative recording. Green arrowheads indicate destaining puncta, white arrowheads non-destaining puncta. (E) Traces of destaining (upper) and non-destaining (lower) puncta shown as mean ± SEM. (F) Quantification of destaining puncta, comparing non-stimulated (No stim) and stimulated (Stim) regions within the same slice. Paired *t* test, n = 4 mice. Scale bar = 5 μm.
  c. Close FIJI, reopen it and run “1-Pearson-time-one_channel.ijm” code again.
  d. Select “No” on step ‘b’ to analyze the second half of the image.
    ***Note:*** The code creates the folder “Data” and will generate two.csv files that will later be used for the correction of the imaging z-drift.
14. In Jupyter Notebook, run the “2-Pearson-FIJI-time.ipynb” code.

Provide the full path of the folder “Data” (Figure 5B). The code merges the two.csv files generated in the previous step.

***Note:*** Full path can be obtained by checking the properties of the folder.

15. Open the “3-Correct-Z-times-3frames_Sum.ijm” code in FIJI.
  a. Confirm that you performed the previous step.
  b. Open the image file “Channel-2.tiff”.
    i. The code will apply z-drift correction routine based on Pearson correlation. Please refer to Methods video S1 for a representative corrected recording.
      Methods Video S1. Final recording originated after step 17 in data analysis, displayed using “fire” lookup table
16. Open the “4-Destain-measure.ijm” code in FIJI.
  a. At prompt, open the file “registered-corrected.tiff”.
  b. The code then asks you to manually curate the ROIs, eliminating any that are at the edges of the registered image, or possible duplicates (Figure 5C).
    ***Note:*** The code performs parallel multi-thresholding with three different algorithms in FIJI. It removes any duplicate ROI and measures the average ROI intensity values.
    **CRITICAL:** ROIs at the edges may end up including areas outside of the image after movement correction. This will create false positives, as well as “NaN” values that interrupt the code.
  c. Select a background ROI.
    **CRITICAL:** The background must not contain any destaining or non-destaining puncta.
17. In Jupyter Notebook, run the “5-Destaining-analysis.ipynb” code.
  a. Provide the full path of the folder with all your data and images (Figure 5C, finishing at the “Image” folder).
    ***Note:*** To identify destaining puncta, the code will first correct for bleaching, then identify puncta that have destained more than 20% of their baseline intensity after electrical stimulation. This roughly corresponds to a 100% increase in destaining compared to the rate of photobleaching.
    i. This code generates the following images as.html.
    all-pres-raw: the individual traces of the boutons without bleach correction.
    all-pres-corrected: the individual traces of the boutons corrected for bleaching.
    Correction-functions: the individual traces of correction functions used for every bouton.
    des_pres: the individual traces of the destaining boutons corrected for bleaching.
    nodes_pres: the individual traces of the non-destaining boutons corrected for bleaching.
    meanall-raw: the trace for all boutons, without bleach correction, represented as mean ± SEM.
    meanall-corrected: the trace for all boutons, corrected for bleaching, represented as mean ± SEM.
    meandes: the trace for all destaining boutons, corrected for bleaching, represented as mean ± SEM.
    meannodes: the trace for all non-destaining boutons, corrected for bleaching, represented as mean ± SEM.
    ii. The code will create a text file titled with the percentage of destaining boutons in the analyzed image.
      ***Note:*** The code uses the data points before electrical stimulation for each punctum and fits a Log_1/2_ curve. For every time point, the code subtracts the value of the fitted curve to the measured data, providing a bleach corrected value.

## Expected outcomes

Live imaging of the dorsal striatum shows stimulation-dependent destaining of FFN200-labelled boutons. This corresponds to boutons capable of DA release. Under physiological conditions, approximately ∼20% of the labelled boutons should destain. This is measured as “ΔF/F_0_” where F_0_ corresponds to the average mean value of each presynapse during the first 20 frames (i.e., at baseline), and ΔF = F- F_0_ where F corresponds to the mean value of each presynapse at a given time point.

From each animal, 6 striatal slices are expected, which can then be hemisected and each hemi-striatum used for an experiment. For every experiment, it is important to have a non-stimulated control. For that reason, we recommend imaging a region adjacent to the selected one for the proper experiment. As Figures 5D–5F shows, electrical stimulation affects the number of detected destaining boutons. The use of bleaching correction provides for the detection of valid destaining. Note that during the imaging, the slices are constantly perfused with ACSF without FFN200. This step washes out some FFN200, which accounts for the negative signal in the non-destaining trace in Figure 5B. However, the effect is small and accounted for in the code.

From these images, users can spatially locate synapses and use this information to run co-localization experiments, for example by labelling lysosomes or mitochondria. Moreover, this protocol can be easily adapted to other FFN techniques such as FFN102^14^ or FFN270,^15^ providing higher temporal resolution.

## Limitations

Imaging other channels at the same time can be challenging. Due to the use of a short wavelength to image FFN200 and the high-power laser settings, it can bleach fluorophores and/or induce photodamage to the tissue. Therefore, the protocol may need some adaptations to perform multi-channel long-term imaging, which may vary between different setups. Parameters that ameliorate bleaching and photodamage risk include decreasing the laser power to image FFN200, decreasing the dwell time, decreasing the number of averaged frames, or imaging for a shorter time duration.

In addition, due to the high magnification used (60x objectives are routinely employed for bouton imaging), any mechanical drifts may compromise the usability of the data.

It is important to consider that two-photon microscopes differ widely between laboratories. Therefore, acquisition parameters and the code used for the analysis may need some adjustments to improve puncta detection (code 4) and calculation of destaining parameters (code 5).

This protocol shows how FFN200 can be used to assess temporal kinetics of DA boutons. However, this can range up to 10s of seconds. Therefore, this can be a potential limitation if faster imaging is required. However, other FFNs can be used to achieve higher temporal resolution, such as FFN102^14^ or FFN270.^15^

FFN200 may be unsuitable for high-throughput imaging due to the long washing time with constant perfusion. However, another VMAT2 substrate, FFN206, has been used for such experiments.^16^^,^^17^^,^^18^

## Troubleshooting

### Problem 1

FFN200 staining is not as bright as in previous experiments (step 8).

### Potential solution

Decrease the washing time or prepare fresh stocks of FFN200. If this happened >5h after animal sacrifice, slice health may have been compromised.

### Problem 2

There is no significant destaining when applying electrical stimulation (step 11-12).

### Potential solution

Always check that the stimulation electrode is working. For example, before placing the electrode in the tissue, bring the tip of the electrode into focus outside of the slice and perform a strong electrical stimulation (e.g., 1 mA, continuous pulsing). If the electrode is working, bubbles will form at the tip. If you still do not observe destaining, you may want to increase the amperage of the stimulation.

### Problem 3

There is too much movement when applying electrical stimulation (step 11-12).

### Potential solution

Two reasons can cause this: (1) the field of view is too close to the tip of the electrode, or (2) the electrical stimulation paradigm is too strong. For (1), image further from the electrode, but within 100 μm of distance. For (2), decrease the amplitude of the stimulation current while increasing the time of the stimulation if necessary. As an option, several trains of stimulation separated by a pause can be used.

### Problem 4

During the image acquisition, the destaining is not happening properly and FFN200 signal dims very quickly (step 12).

### Potential solution

Two reasons that this can occur: (1) high photobleaching is happening, or (2) the slice is unhealthy or dead. For (1), try a gentler laser intensity or faster scanning time. For (2), try decreasing the time between slice preparation and imaging. If unsure, propidium iodide staining of dead cells can be used as a guide.

### Problem 5

There is too much drift throughout image acquisition, independent of electrical stimulation (step 11-12).

### Potential solution

Two reasons can cause this: (1) the perfusion is too fast, or (2) the incubation time in the imaging chamber is too short. For (1), decrease the perfusion speed, 1-2 mL/min is enough to keep the slice alive while imaging. For (2), increase the incubation time in the imaging chamber to let the system settle.

## Resource availability

### Lead contact

Further information and requests for resources and reagents should be directed to and will be fulfilled by the lead contact, David L. Sulzer (ds43@cumc.columbia.edu).

### Technical contact

Technical questions on executing this protocol should be directed to and will be answered by the technical contact, Patrick Cottilli (patrick.cottilli@crick.ac.uk).

### Materials availability

This study did not generate any new unique reagents.

### Data and code availability

All original code has been deposited at GitHub and Figshare. This is publicly available at Github: https://github.com/MDevineLab/FFN200-live-imaging and Figshare: https://doi.org/10.25418/crick.32731767. We also provide an example of original raw data for users.

## Acknowledgments

This work was supported by The 10.13039/100010438Francis Crick Institute (P.C. and M.J.D.), which receives its core funding from 10.13039/501100000289Cancer Research UK (CC2206), the 10.13039/501100000265UK Medical Research Council (CC2206), and the 10.13039/100010269Wellcome Trust (CC2206). This work was supported by FTF Foundation and 10.13039/100000002NIH 10.13039/100000026NIDA 07418 (D.L.S.). P.C. was also supported by a Bogue Fellowship from 10.13039/501100000765University College London and The Harold Hyam Wingate Foundation Medical Research travel grant. We thank Sejoon Choi for slice-preparation support. We thank Ryan Dosumu-Johnson, Mark Sonders, Xavier Westergaard, Siham Boumhaouad, and Daniela Pereira for imaging support. We thank Vanessa Morales for supporting animal work. We thank Jonathan I. Spencer for photographic assistance. The references for figures were done in BioRender: https://BioRender.com/ltgpsd3.

## Author contributions

P.C.: investigation, methodology, formal analysis, software, funding acquisition, and writing – original draft; M.J.D.: supervision, resources, funding acquisition, and writing – original draft; E.V.M.: conceptualization, supervision, investigation, and writing – original draft; D.L.S.: conceptualization, supervision, funding acquisition, and writing – original draft.

## Declaration of interests

The authors declare no competing interests.
